# *Pseudomonas aeruginosa* NfsB and nitro-CBI-DEI – a promising enzyme/prodrug combination for gene directed enzyme prodrug therapy

**DOI:** 10.1186/1476-4598-12-58

**Published:** 2013-06-10

**Authors:** Laura K Green, Sophie P Syddall, Kendall M Carlin, Glenn D Bell, Christopher P Guise, Alexandra M Mowday, Michael P Hay, Jeffrey B Smaill, Adam V Patterson, David F Ackerley

**Affiliations:** 1 School of Biological Sciences, Victoria University of Wellington, Kelburn Parade, Wellington, New Zealand; 2 Auckland Cancer Society Research Centre, University of Auckland, Grafton, Auckland, New Zealand; 3 Maurice Wilkins Centre for Molecular Biodiscovery, School of Biological Sciences, University of Auckland, Auckland, New Zealand; 4 Centre for Biodiscovery, School of Biological Sciences, Victoria University of Wellington, Wellington, New Zealand

**Keywords:** Gene therapy, GDEPT, Nitroaromatic prodrug, Nitroreductase, Nitro-CBI-DEI, CB1954, SOS chromotest, Bystander effect

## Abstract

**Background:**

The nitro-chloromethylbenzindoline prodrug nitro-CBI-DEI appears a promising candidate for the anti-cancer strategy gene-directed enzyme prodrug therapy, based on its ability to be converted to a highly cytotoxic cell-permeable derivative by the nitroreductase NfsB from *Escherichia coli*. However, relative to some other nitroaromatic prodrugs, nitro-CBI-DEI is a poor substrate for *E. coli* NfsB. To address this limitation we evaluated other nitroreductase candidates from *E. coli* and *Pseudomonas aeruginosa*.

**Findings:**

Initial screens of candidate genes in the *E. coli* reporter strain SOS-R2 identified two additional nitroreductases, *E. coli* NfsA and *P. aeruginosa* NfsB, as being more effective activators of nitro-CBI-DEI than *E. coli* NfsB. In monolayer cytotoxicity assays, human colon carcinoma (HCT-116) cells transfected with *P. aeruginosa* NfsB were >4.5-fold more sensitive to nitro-CBI-DEI than cells expressing either *E. coli* enzyme, and 23.5-fold more sensitive than untransfected HCT-116. In three dimensional mixed cell cultures, not only were the *P. aeruginosa* NfsB expressing cells 540-fold more sensitive to nitro-CBI-DEI than pure cultures of untransfected HCT-116, the activated drug that they generated also displayed an unprecedented local bystander effect.

**Conclusion:**

We posit that the discrepancy in the fold-sensitivity to nitro-CBI-DEI between the two and three dimensional cytotoxicity assays stems from loss of activated drug into the media in the monolayer cultures. This emphasises the importance of evaluating high-bystander GDEPT prodrugs in three dimensional models. The high cytotoxicity and bystander effect exhibited by the NfsB_Pa/nitro-CBI-DEI combination suggest that further preclinical development of this GDEPT pairing is warranted.

## Background

In gene-directed enzyme prodrug therapy (GDEPT) tumour cells are sensitised, via selective transgene delivery and/or expression, to a systemically administered prodrug. A key aspect of GDEPT is the bystander effect, the ability of activated prodrugs to transport either passively or actively out of the cell of origin and into neighbouring non-transfected cells, which provides an elegant solution to the unavoidable issue of low cell transfection rates [1]. Studies employing prodrugs with high bystander effects have demonstrated that significant tumour reduction can occur when less than 0.1% of the tumour population expresses the activating enzyme [2].

**Figure 1:**
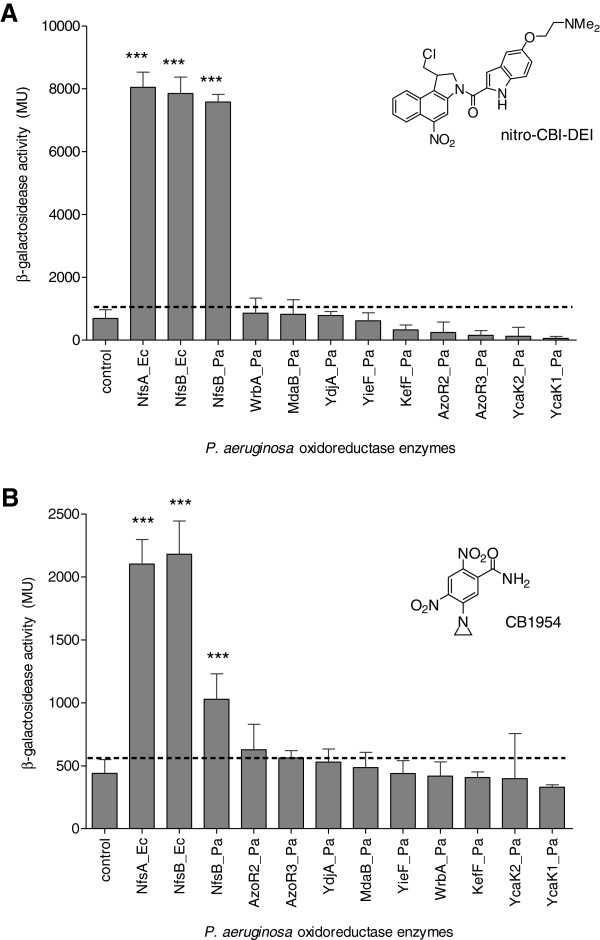
**Fold-induction of SOS response in SOS-R2 strains over-expressing candidate nitroreductases upon challenge with CB1954 or nitro-CBI-DEI.** Mid-exponential phase cultures of each SOS-R2 *sfiA:lacZ* reporter strain, either over-expressing a candidate nitroreductase from the expression vector pUCX or containing an empty pUCX plasmid control, were incubated for 3 h in the presence of **A**. 5 μM nitro-CBI-DEI or **B**. 20 μM CB1954, after which relative induction of the SOS response in each strain was measured by β-galactosidase assay as previously described [8]. Data are the mean Miller units from three independent experiments, each performed in duplicate; and error bars are ± 1 standard deviation. The black dotted line indicates the basal SOS activity in the empty plasmid control. *** indicates p < 0.001 by one-way ANOVA with Dunnett comparison of test to control.

Bacterial type I nitroreductase enzymes, which catalyse the simultaneous two-electron bioreductive activation of nitroaromatic prodrug substrates, hold great potential for GDEPT. To date, the majority of nitroreductase GDEPT studies have focused on the prodrug CB1954 [5-(aziridin-1-yl)-2,4-dinitrobenzamide] (Figure 1B, structure inset), which exhibits only a modest bystander effect upon activation [3]. However, the intrinsically oxygen insensitive nature of the two-electron reduction mechanism enables nitroaromatic prodrugs that were originally designed to target tumour hypoxia (i.e. by exploiting the oxygen-sensitive one-electron reduction mechanism of endogenous human reductases), to potentially be re-purposed for nitroreductase GDEPT [1]. The main class of hypoxia-activated prodrugs to have been considered in this context is the dinitrobenzamide mustards (DNBMs) e.g. [4-6]; not only are these substantially more cytotoxic and generally better tolerated than CB1954, they also typically exert a greater bystander effect [3,4,7].

Also promising for GDEPT are the nitro-chloromethylbenzindolines (nitro-CBIs), originally designed to be hypoxia-activated prodrugs [9] of amino analogues of the cyclopropylindoline anti-tumour antibiotics, exemplified by CC-1065 and duocarmycin SA [10,11]. It has been shown that the *Escherichia coli* nitroreductase NfsB (NfsB_Ec) can reduce nitro-CBIs in an oxygen-independent fashion, generating highly cytotoxic metabolites that alkylate the N3 of adenine in the minor groove of DNA [12]. However, it was inferred that the lead nitro-CBI prodrug in that study, nitro-CBI-5-[(dimethylamino)ethoxy]indole (nitro-CBI-DEI; Figure 1A, structure inset) is a poor substrate for NfsB_Ec relative to CB1954 or the DNBMs [12]. In this work we sought to identify more active nitroreductases for metabolism of nitro-CBI-DEI, reasoning that superior enzymes will be required to extend the therapeutic index of nitro-CBI prodrugs in GDEPT.

## Findings

To identify novel nitroreductase enzymes we previously constructed an over-expression library of eleven candidate oxidoreductases from *E. coli*[13]. Here, preliminary screens of this library indicated that in addition to NfsB_Ec, *E. coli* NfsA (NfsA_Ec) was able to activate nitro-CBI-DEI to a measurable extent (not shown). We next tested an orthologous collection of oxidoreductase enzymes from *Pseudomonas aeruginosa* (Table 1), which we had independently assembled to investigate their hypothesised role in reducing antioxidant quinones in this bacterium (LK Green, AC la Flamme and DF Ackerley, unpublished work). When NfsA_Ec, NfsB_Ec and the *P. aeruginosa* enzymes were individually over-expressed in the *E. coli* strain SOS-R2, which contains a *lacZ* reporter gene under control of the SOS responsive promoter *sfiA*[6], NfsA_Ec, NfsB_Ec, and NfsB_Pa all generated a powerful induction of the SOS (DNA damage repair) response following challenge with 5 μM nitro-CBI-DEI (Figure 1A). In contrast, only NfsA_Ec and NfsB_Ec induced a substantial SOS response following challenge with 20 μM CB1954 (Figure 1B).

**Table 1:** Nitroreductase candidates evaluated in this study

**Protein name**^**a**^	**Gene locus**^**b**^	**% identity**^**c**^	**Accession no.**^**d**^
NfsB_Pa	PA5190	25 (NfsB_Ec)	AAG08575.1
AzoR2_Pa	PA1962	41 (AzoR_Ec)	AAG05350.1
AzoR3_Pa	PA3223	45 (AzoR_Ec)	AAG06611.1
KefF_Pa	PA4975	37 (KefF_Ec)	AAG08360.1
MdaB_Pa	PA2580	64 (MdaB_Ec)	AAG05968.1
WrbA_Pa	PA0949	39 (WrbA_Ec)	AAG04338.1
YcaK1_Pa	PA1225	33 (YcaK_Ec)	AAG04614.1
YcaK2_Pa	PA0853	27 (YcaK_Ec)	AAG04242.1
YdjA_Pa	PA3208	42 (YdjA_Ec)	AAG06596.1
YieF_Pa	PA1204	45 (YieF_Ec)	AAG04593.1
NfsA_Ec	-	100 (NfsA_Ec)	BAA35562.1
NfsB_Ec	-	100 (NfsB_Ec)	AAC73679.1

^a^Protein nomenclature as assigned in this study, based on existing nomenclature of closest *E. coli* orthologue, with the exception of AzoR2 and AzoR3 named as per existing GenBank annotations. Suffix _Pa indicates host of origin was *P. aeruginosa* PAO1; _Ec indicates *E. coli* W3110.

^b^Gene locus ID for *P. aeruginosa* proteins, as assigned in Pseudomonas Genome Database [14].

^c^Percentage amino acid identity shared with closest *E. coli* orthologue (identified in parentheses).

^d^GenBank accession number for protein sequences.

**Figure 2:**
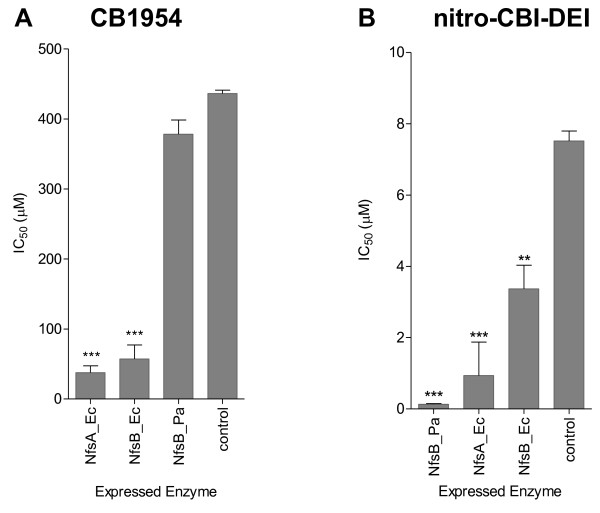
**Bacteria-delivered enzyme prodrug cytotoxicity assay.** Calculated IC_50_ of HCT-116 cells post-incubation with *E. coli* cells (over-expressing either NfsA_Ec, NfsB_Ec, NfsB_Pa, or an empty pUCX control) across a serial 2-fold dilution series of **A**. CB1954 (25 μM to 400 μM); or **B**. nitro-CBI-DEI (0.06 μM to 15 μM). Following 4 h of incubation bacteria and prodrug were removed by PBS washes, and surviving HCT-116 cells allowed to recover in fresh Dulbecco's modified Eagle's medium supplemented with fetal calf serum, 10 mM HEPES and 100 μg/ml chloramphenicol, as described [8]. Percentage cell survival at each prodrug concentration was then calculated relative to an unchallenged control by CellTiter 96^®^ AQueous One Solution Cell Proliferation Assay (Promega). Data are the mean of three independent experiments, each performed in duplicate; and error bars are ± 1 standard deviation. ** indicates p < 0.005 and *** p < 0.001 by one-way ANOVA with Dunnett comparison of test to control.

To further distinguish the ability of NfsA_Ec, NfsB_Ec, and NfsB_Pa to activate each prodrug we employed a bacteria-delivered enzyme cytotoxicity assay, as previously described [8]. *E. coli* strains individually over-expressing each nitroreductase or containing an empty plasmid control were incubated in co-culture with a monolayer of untransfected human colon carcinoma (HCT-116) cells across a range of concentrations of CB1954 (25–400 μM) or nitro-CBI-DEI (0.06-15 μM). Consistent with the SOS assays, the two *E. coli* enzymes were far more effective than NfsB_Pa in sensitizing HCT-116 cells to CB1954 (Figure 2A); whereas NfsB_Pa exhibited a significantly lower IC_50_ (the concentration of prodrug required to inhibit growth of HCT-116 to 50% of unchallenged control levels) with nitro-CBI-DEI than either NfsA_Ec or NfsB_Ec (p ≤ 0.001; T-test) (Figure 2B).

**Figure 3:**
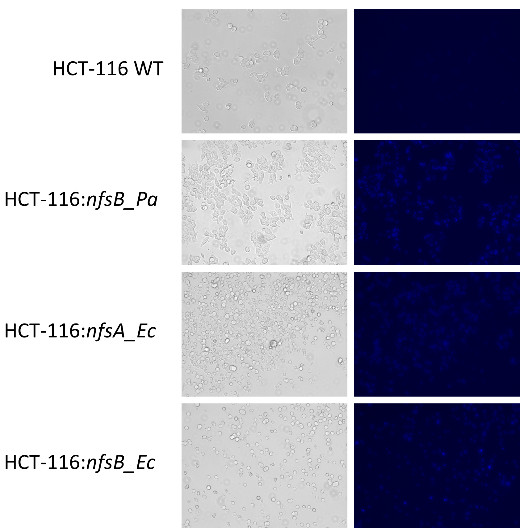
**Confirmation of functional nitroreductase expression in HCT-116 cells using the fluorogenic probe FSL81.** FSL81 is metabolised to a blue fluorescent product in HCT116 cells expressing *nfsB_Pa*, *nfsA_Ec* or *nfsB_Ec*, but not in untransfected wild type (WT) cells. Cells were plated at a density of 50,000 cells per well in a 24 well plate containing fresh αMEM media amended with 5% fetal calf serum. After 3 h media was aspirated and replaced with fresh media containing 50 μM FSL81, followed by incubation for a further 2 h. Images are relief phase (left) or fluorescence mode (right; excitation 390/40 nm, emission 446/33 nm) taken using an EVOS^®^ Floid^®^ Cell Imaging Station (Invitrogen, Carlsbad, CA).

We next created HCT-116 cell lines stably transfected with NfsA_Ec, NfsB_Ec or NfsB_Pa, using the Gateway™ compatible expression plasmid F527-V5, which expresses inserted genes from a constitutive human elongation factor-1 alpha promoter (all details as per [13]). Functional nitroreductase activity was confirmed qualitatively for all three stably transfected cell lines, but not the parental HCT-116 cells, using the bacterial nitroreductase specific fluorogenic probe FSL81 as previously described [6] (Figure 3). The sensitivity of each cell line towards nitro-CBI-DEI was then evaluated using an *in vitro* proliferation assay as described [3]. Briefly, replicate monolayers (n = 3) of NfsA_Ec, NfsB_Ec, NfsB_Pa or non-transfected HCT-116 cells were exposed to a range of concentrations of nitro-CBI-DEI (dilution series from 15.2 pM to 0.3 μM) for 18 h. Cells were washed free of drug and incubated for a further 4 days, after which wells were stained with sulforhodamine B to detect protein as a measure of cell proliferation. Consistent with the results of the bacteria-delivered cytotoxicity assays, the stably transfected NfsB_Pa cell line (IC_50_ = 2.0 ± 0.4 nM) was significantly more sensitive to nitro-CBI-DEI than either the NfsA_Ec (IC_50_ = 24 ± 5 nM), NfsB_Ec (IC_50_ = 9.0 ± 1.2 nM) or parental HCT-116 (IC_50_ = 47 ± 9.2 nM) cell lines (p ≤ 0.003; T-test). The 5.2-fold differential between WT and NfsB_Ec cells is consistent with the 4.6-fold differential reported previously for these cell lines [12] but is considerably smaller than the 23.5-fold differential observed here between WT and NfsB_Pa cells.

**Figure 4:**
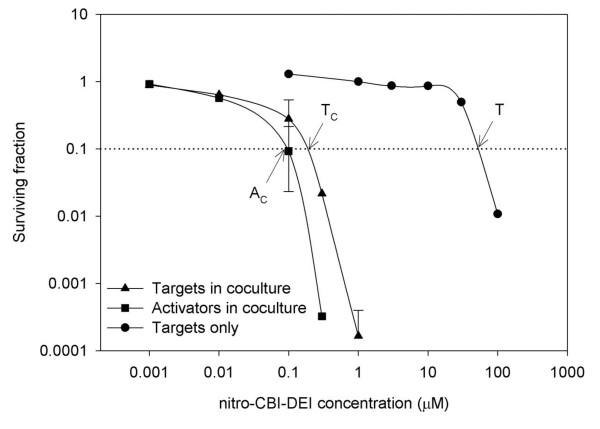
**Bystander effect from metabolic activation of nitro-CBI-DEI in multicellular layer cultures.** Clonogenic survival curves were generated using cells from three-dimensional multicellular layers exposed to nitro-CBI-DEI under hyperoxic conditions (95% O_2_; used to avoid hypoxia/necrosis in cells at the centre together with confounding activation of nitro-CBI-DEI due to hypoxia). Multicellular layers consisted of 100% HCT116 wild type ‘target’ cells or cocultures of target cells in the presence of a small percentage of HCT116 NfsB_Pa ‘activator’ cells (5.8 ± 3.2%). To establish the C_10_ values of target and activator cells in co-culture, cells were plated in both non-selective and selective media (the latter amended with 3 μM puromycin). The target cell population was calculated by subtracting the number of puromycin resistant activator cells (colonies counted on selective plates) from the total cell population (colonies counted on non-selective plates). Points are the combined data sets from two independent experiments with overlapping data points; and error bars are ± 1 standard deviation. The dashed line represents one log of cell kill. The C_10_ values in targets alone (T), targets in co-culture (T_C_) or activators in co-culture (A_C_) were interpolated.

The role of NfsB_Ec in the activation of nitro-CBI-DEI has previously been explored in a three dimensional cell culture model, in which expression of NfsB_Ec sensitised HCT-116 cells to nitro-CBI-DEI by a factor of 12-fold, and there was evidence of a substantial bystander effect for the activated metabolite(s) [12]. Having shown NfsB_Pa to be superior to NfsB_Ec at activating nitro-CBI-DEI in cell monolayers, we next sought to quantify the ability of a minority of NfsB_Pa transfected ‘activator’ cells to kill untransfected HCT-116 ‘target’ cells in three dimensional (3D) co-cultures [3], via the bystander effect of activated nitro-CBI-DEI metabolites (Figure 4). Sensitivity to nitro-CBI-DEI was calculated using a post-exposure clonogenic endpoint [3], where the C_10_ value represents the concentration of nitro-CBI-DEI that yields one log of cell kill. In cultures of 100% target cells, the C_10_ value was 52.3 μM (Figure 4). In contrast, in co-cultures of target cells and a minority (5.8% ± 3.2%) of NfsB_Pa-transfected activator cells, the activator cells were 540-fold more sensitive to nitro-CBI-DEI, with a C_10_ value of 0.097 μM (Figure 4). The target cells in this 3D co-culture were nearly as sensitive as the activators, their C_10_ value of 0.19 μM indicating an almost perfectly uniform transfer of toxicity (Figure 4). The overall bystander effect efficiency (BEE) was calculated to be 89%; this strikingly efficient transfer of cytotoxicity compares to previously measured BEEs of 13% for CB1954, and 48% for the lipophilic DNBM prodrug SN27686 (using NfsB_Ec expressing activator cells) [4]. The BEE is a measure of the extent to which activator cells cause the dose response curve for co-cultured target cells to shift toward the activator dose response curve; and is calculated according to the formula (LogC_10_T-LogC_10_T_C_)/(LogC_10_T-LogC_10_A_C_), where C_10_T is the C_10_ value for a pure culture of target cells, C_10_T_C_ is the C_10_ value for target cells in co-culture with activators, and C_10_A_C_ is the C_10_ value for those activator cells [4]. It is important to acknowledge that the quoted BEEs for CB1954 and SN27686 were measured using only a 1% activator cell population [4]. However, in another study that used a 50% activator cell population [7], the shift in dose response curve for the co-cultured target cells was still less substantial with any of the five prodrugs examined (CB1954, RSU-1069, triapazamine, or the DNBMs SN23862 or SN23816) than with nitro-CBI-DEI and the 5.8% activator cell population in our study.

The disparity between killing of NfsB_Pa transfected HCT-116 cells due to nitro-CBI-DEI activation in the *in vitro* IC_50_ proliferation assay and the 3D mixed cell cultures highlights the importance of evaluating potential GDEPT enzyme-prodrug partnerships in a 3D bystander model, as low cell density may dramatically underestimate cytotoxic potential. We surmise that the cytotoxic potential of nitro-CBI-DEI may be dramatically underestimated in low cell density proliferation assays due to washout and loss in the media, as suggested for other high-bystander prodrugs [3].

The unprecedented level of bystander killing, coupled with the substantial superiority of NfsB_Pa over NfsB_Ec in sensitising 3D cultures of nitroreductase-expressing HCT-116 cells to nitro-CBI-DEI, suggests that the NfsB_Pa/nitro-CBI-DEI combination is worthy of further evaluation in preclinical GDEPT models. Should it prove necessary to further enhance enzyme activity via directed evolution or targeted mutagenesis studies, our demonstration that nitro-CBI-DEI induces the *E. coli* SOS response upon activation indicates that it should be possible to recover improved NfsB_Pa variants by SOS screening, as previously used to generate superior CB1954 activating variants of the *Vibrio fischeri* nitroreductase FRaseI [8].

## Competing interests

The authors declare that they have no competing interests.

## Authors’ contributions

LKG generated the *P. aeruginosa* oxidoreductase gene library and performed all experiments involving *E. coli* strains, with guidance from DFA. SPS, KMC and GB performed all experiments involving nitroreductase transfected HCT-116 cells, with guidance from AVP. MPH and JBS prepared the chemical library from which the prodrugs were obtained and contributed to study design. AMM and CPG played key roles in data interpretation and figure preparation. DFA wrote the manuscript, with contributions from LKG, AMM, and CPG. All authors read and approved the final manuscript.
